# The dahliagram: An interdisciplinary tool for investigation, visualization, and communication of past human-environmental interaction

**DOI:** 10.1126/sciadv.adj3142

**Published:** 2023-11-22

**Authors:** Michael Frachetti, Nicola Di Cosmo, Jan Esper, Lamya Khalidi, Franz Mauelshagen, Clive Oppenheimer, Eleonora Rohland, Ulf Büntgen

**Affiliations:** ^1^ Department of Anthropology, Washington University in St. Louis, 1 Brookings Drive, CB 1114, St. Louis, MO 63130, USA.; ^2^ School of Cultural Heritage, Northwest University, Xi’an, China.; ^3^ Institute for Advanced Study, Princeton University, Princeton, NJ 08544, USA.; ^4^ Department of Geography, Johannes Gutenberg University, Becherweg 21, 55099 Mainz, Germany.; ^5^ Global Change Research Institute (CzechGlobe), Czech Academy of Sciences, 603 00 Brno, Czech Republic.; ^6^ Université Côte d’Azur, CNRS, CEPAM, 24 avenue des Diables Bleus, 06300 Nice, France.; ^7^ Department of Social Anthropology, University of Bielefeld, 33615 Bielefeld, Germany.; ^8^ Department of Geography, University of Cambridge, Cambridge CB2 3EN, UK.; ^9^ Department of History, University of Bielefeld, 33615 Bielefeld, Germany.; ^10^ Swiss Federal Research Institute (WSL), 8903 Birmensdorf, Switzerland.; ^11^ Department of Geography, Faculty of Science, Masaryk University, 613 00 Brno, Czech Republic.

## Abstract

Investigation into the nexus of human-environmental behavior has seen increasing collaboration of archaeologists, historians, and paleo-scientists. However, many studies still lack interdisciplinarity and overlook incompatibilities in spatiotemporal scaling of environmental and societal data and their uncertainties. Here, we argue for a strengthened commitment to collaborative work and introduce the “dahliagram” as a tool to analyze and visualize quantitative and qualitative knowledge from diverse disciplinary sources and epistemological backgrounds. On the basis of regional cases of past human mobility in eastern Africa, Inner Eurasia, and the North Atlantic, we develop three dahliagrams that illustrate pull and push factors underlying key phases of population movement across different geographical scales and over contrasting periods of time since the end of the last Ice Age. Agnostic to analytical units, dahliagrams offer an effective tool for interdisciplinary investigation, visualization, and communication of complex human-environmental interactions at a diversity of spatiotemporal scales.

## Teaser

Dahliagrams offer interdisciplinary investigation and visualization of human-environmental interactions at diverse scales.

## INTRODUCTION

Bringing evidence of environmental and climatic changes into discourse for understanding human history and behavior is not new (*1*). However, recent years have seen more concerted efforts to promote consilience through dialogue between the sciences and humanities (*2*, *3*), as well as the application of genetics to questions of past human geography and demography (*4*, *5*). Such studies are generally published in scientific journals (*6*–*9*), arguably limiting their influence on historians for whom monographs and a small number of history journals remain vital academic currency (*10*–*13*). Multidisciplinary studies of the entanglements between climate and society, generally, have also not been without their critics (*14*), and there is a thin line between over- and underinterpretation of direct and indirect linkages between human behavior and environmental factors.

With increasingly refined proxy reconstructions and model simulations of Holocene climates (*15*), as well as new insights into human organization and mobility during the past millennia (*16*, *17*), there are now rich opportunities to explore the interplay of social, political, economic, and environmental factors on human behavior through time and space. Yet, the current pivot toward “small-scale” case studies that leverage high-resolution climate proxy records can have an inadvertent effect of being too narrow or idiosyncratic to provide effective explanations of more general trends throughout antiquity (*18*). Interdisciplinary attempts to engage data from history, climate science, archaeology, and ecology to understand past socio-environmental interactions often face mismatched metrics, inspiring new approaches that aim to compile, corelate, and visualize diverse lines of evidence at a range of scales and uncertainties in robust scientific manner (*3*, *19*). While such methods allow for productive conceptual cross-assessment and insight, they require a comparative and qualitative tool to standardize the varied scales and resolutions of data and diverse research sources used to explain complex socioenvironmental behaviors, such as population movement.

Today, more than 280 million people or roughly 4% of the world’s population live outside their country of birth (*20*). Despite decades of intensive research concerning the overlapping socioeconomic and environmental factors that may motivate or force human mobility (*21*), explanations of (pre)historic (and modern) population movements often remain restricted to monocausal explanations, such as climate, conflict, or economy (*22*). Overly simplistic views, however, can create a circular narrative between the causes and consequences of movement and the practices and policies proposed as sustainable solutions (*23*). Given the heightened attention to the implication of past climate variation for historic human migration (among other socioenvironmental behaviors), we here offer an interdisciplinary tool to visualize the relative roles of environmental factors alongside social, political, and economic influences. Moreover, we assess the impacts of a range of factors on local- to large-scale mobility in three regional settings throughout the past ~12,000 years.

## MATERIALS AND METHODS

### The dahliagram for interdisciplinary investigation

**Fig. 1.**
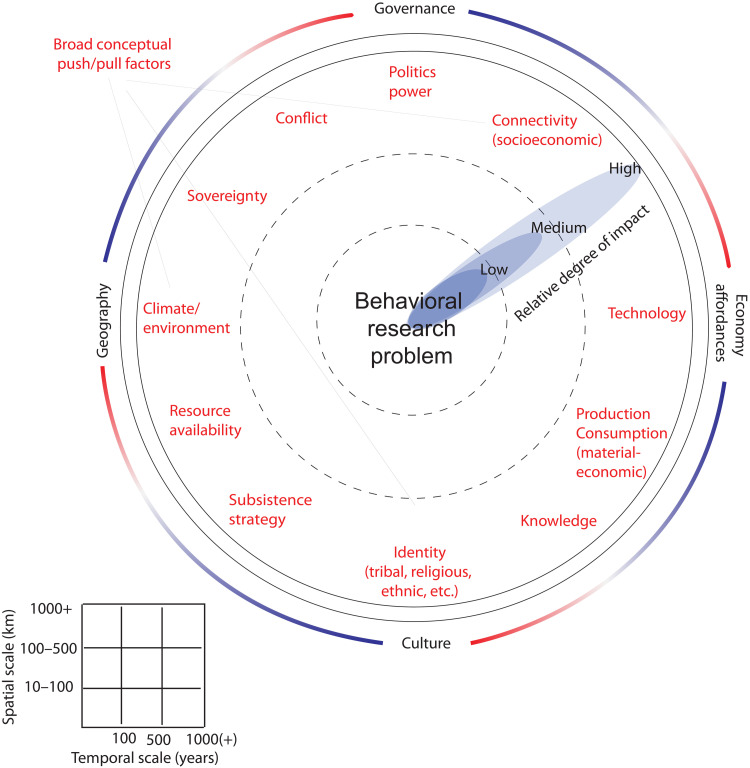
Schematic template of the dahliagram. Push/pull factors are arranged in a ring surrounding the central research problem. Petal length for each factor is ranked at low, medium, or high on the basis of interdisciplinary assessment. Analytical scale is indicated in the lower left according to both spatial and temporal ranges considered.

The dahliagram represents a qualitative tool for synthesizing and visualizing knowledge drawn from a wide range of disciplines and spatiotemporal scales (Fig. 1). Although the dahliagram is a universal device for socioenvironmental research, we focus on human mobility as a behavioral response in three pivotal regions of world history: eastern Africa, Inner Eurasia, and the North Atlantic. Outlined for select phases of pronounced mobility since the end of the last Ice Age, the resulting dahliagrams represent easily accessible and customizable visualizations that enable qualitative comparison of the relative influences of diverse social and environmental factors on human behavior.

In this study, “movement” is placed at the center of the dahliagram while different push and pull factors are represented in a surrounding array of “petals.” Each factor is evaluated on the basis of interdisciplinary synthesis of available research and ranked according to its influence from low to high, charted over three concentric rings of increasing intensity. Factor categories can be customized depending on the analytical focus, although they should be sufficiently broad to accommodate shifting definitions over time and space.

For example, “technology” might refer to the emergence of novel materials, such as bronze (in deeper antiquity) or the improvement of sailing vessels (in the 15th century CE) each of which influenced human movements in their own contextual setting. In our case, dahliagrams also accommodate diverse data sources and scholarly traditions that shape how knowledge is gathered and used to weight the impact of factors on human movements. By considering and rating multiple factors in a single image, comparable to the concept of planetary boundaries (*24*), the intersectional social and environmental framework of a dahliagram stimulates conceptual thinking and promotes critical engagement with the corollary conditions surrounding complex human-environmental feedbacks, including climate. When applied diachronically within a regional context, a time series of dahliagrams can reveal phased transitions within and between various socioenvironmental factors, encouraging specialized research into one or more of the pertinent domains.

The selected regional case studies for our three dahliagrams range in chronological scale from decades to centuries to millennia, framed according to historical or archaeological documentation of human movement in each regional case (indicated in the lower-left table of each dahliagram). Throughout our group’s ongoing interdisciplinary interactions, we have found dahliagrams to be as effective in assessing population movements that occurred within richly documented historical timescales as they are for modeling more protracted periods of human mobility, because in both cases, the social and environmental factors can be qualitatively ranked according to the available resolution of their underlying data, without requiring a common unit of quantification. The dahliagram is particularly effective when considering lower-resolution archaeological data alongside more-detailed climate data because it facilitates the interpolation of diverse data sources within one visualization. Its graphical layout intentionally positions the relevant factors with equal potential to influence the behavior in question, although each factor can be ranked independently (as “low,” “medium,” or “high”) according to its quantitative and/or qualitative measurements. Of course, applications of the dahliagram should carefully consider and make transparent questions related to data resolution, archival composition, and other source-related issues, depending on the evidential material relevant to each case.

## RESULTS

### Human movement in eastern Africa and southern Arabia

**Fig. 2.**
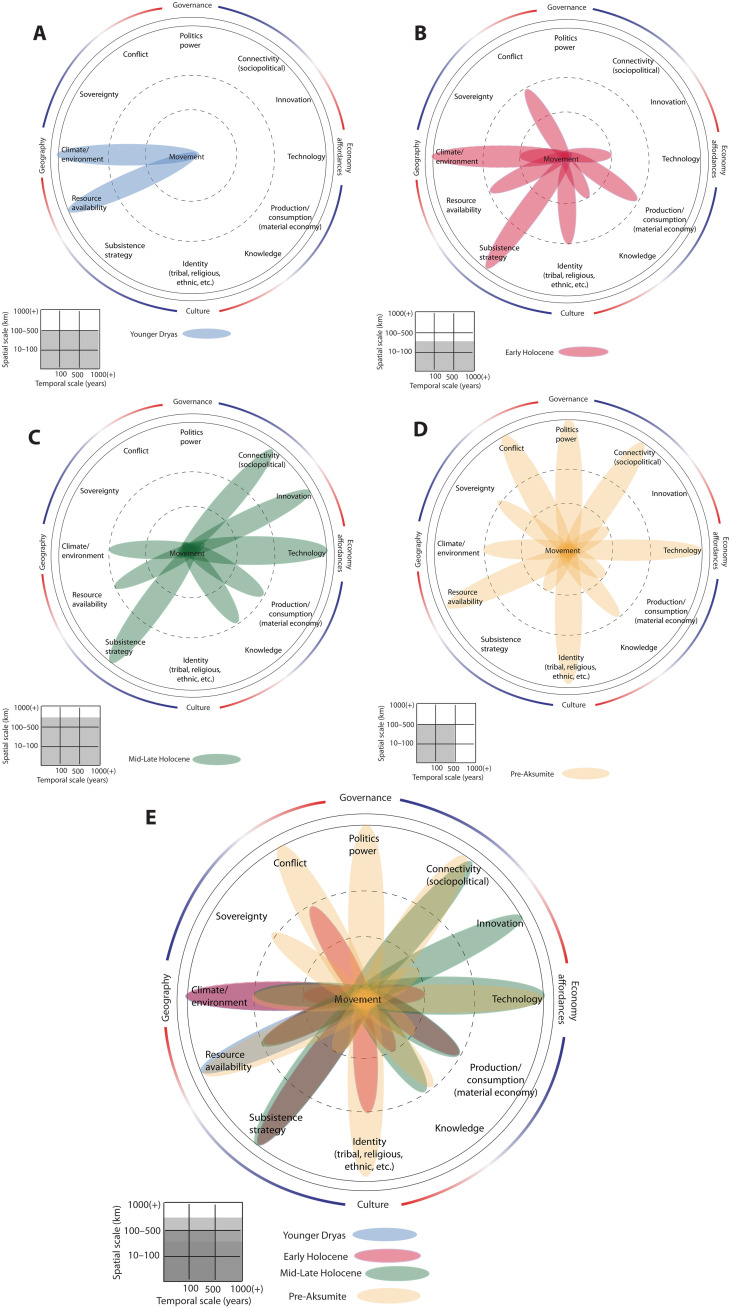
Dahliagram analyses of human movement in east Africa/southern Arabia. (**A**) Younger Dryas (YD) [~12,900 to 11,700 before present (B.P.)], (**B**) Early Holocene (~11,700 to 8200 B.P.), (**C**) Late Holocene (~8200 to 3000 B.P.), (**D**) pre-Aksumite period (~2800 to 2050 B.P. ), and (**E**) multiperiod composite dahliagram for east Africa.

The Horn of Africa and southern Arabia are regions whose deep and recent pasts are inextricably tied to population movement. Mobility has most likely been central to a range of behaviors and conditions that shaped the regions’ extensive linguistic, ethnic, and genetic diversity. “Out of Africa” migrations of hominins including *Homo sapiens* were followed by “back to Africa” movements throughout the Pleistocene and Holocene, evident from both paleogenetic and archaeological records (*25*–*28*). There is robust evidence for human occupation before and after the Younger Dryas (YD), a hyperarid period lasting from ~12,900 to 11,700 years before present (B.P.) (*29*, *30*). Hence, it has been suggested that Late Pleistocene populations moved to refugia in well-watered highlands, coastal plains, and oases (*31*, *32*). These factors suggest that mobility (into refugia) was driven largely by extreme and rapid climatic shifts occurring on the order of centuries (*33*) and geographically focused by access to water and food (Fig. 2A).

The Early Holocene (~11,700 to 8200 B.P.) is marked by the resurgence of the African Humid Period or Holocene Pluvial after the YD event. The increased precipitation reactivated hydrological systems and substantially raised lake levels. It is also thought to have facilitated movement across the deserts of Arabia and the (Green) Sahara while serving to isolate other populations because of abundant resource availability around newly developed lakes and the flooding of major corridors of migration such as the Nile valley and East African Rift (*30*, *34*).

In southern Arabia, this period is marked by resource abundance and persistence of hunter-gatherer-fisher subsistence (*35*). The Early Holocene is characterized by lithic traditions distinct from those found in the Horn of Africa (*36*, *37*). Mobility was still heavily influenced by subsistence strategies, such as fishing, hunting, or foraging. Given the increased availability of food resources at short range, long-distance mobility was likely associated with search for lithic resources (Fig. 2B), such as obsidian.

Major social changes took place in the Middle to Late Holocene (~8200 to 3000 B.P.). Economic transitions toward pastoralism occurred gradually and episodically, first in Yemen and Sudan at the Early-Middle Holocene transition and then in the Horn of Africa toward the end of the Middle Holocene (between 5000 and 4300 B.P.), with certain areas transitioning later. While the Middle Holocene is characterized by the persistence of lakes and perennial rivers in much of Arabia and eastern Africa, the end of the African Humid Period brought aridification between 4.5 and 4.2 B.P. (*30*). In both regions, climate and environment influenced mobility, though to a lesser extent than in earlier periods (Fig. 2C).

In eastern Africa, the transition to herding occurred at the end of the Middle Holocene, between 5000 and 3000 cal B.P. (*30*, *38*, *39*) coinciding with major technological changes including the development of local ceramic traditions, new storage, and food production techniques (ovens, grindstones, etc.) and backed pieces made on flakes rather than bladelets. Cattle, sheep, and goat were adopted into existing foodways that still drew on fishing, hunting, and foraging (*40*).

By the Middle Holocene in Arabia, herding of cattle, sheep, and goats was well established, while foraging and hunting remained common (*41*). Herding posed new challenges for populations, requiring new mobility patterns distinct from those used by hunter-gatherers and social ties becoming invaluable as pasturage and water holes became a routine need for pastoralists (*42*).

The change and increase in distances covered by populations is evident through obsidian sourcing. In southern Arabia, obsidian from the Yemen highlands, as well as other materials such as jade, were found as far as the Hadramawt, western Oman, and southern Saudi Arabia with the onset of the Middle Holocene (*43*, *44*). At about the same time on the Red Sea coast of Yemen, exogenous obsidian replaced local cherts used to make bifacial points, suggesting Red Sea crossings and long-distance exchange. This implies the development of new kinship ties and technologies. By ~5000 BP, these interactions led to African obsidian being brought over to the Red Sea shores of Yemen in increasingly large quantities, along with a new tool type made in the same way as in the African Horn (*45*, *46*). Technology, innovation, connectivity, and new subsistence strategies are therefore the most influential factors for mobility during this time, eclipsing prior push factors such as food resource availability and environment as populations became more dependent on social networks and exchange for resources with outside groups (Fig. 2C) (*47*).

The Iron Age ushered novel political structures in eastern Africa along with the rise of kingly political hegemonies, specialized production workshops, and formalized trade partnerships among distant kingdoms. By 2900 B.P., several Sabaean kingdoms were vying for power and control of the incense trade routes in southern Arabia and had developed water storage systems for agricultural use in arid zones (*48*).

In the Horn of Africa, the same processes were in play during the pre- and Aksumite periods. While throughout the pre-Aksumite period, there was demographic continuity with populations that preceded, Sabaean elements from the Daʿmat polity intermixed with local innovations, eventually leading to a Semitic writing system (Ge’ez), a pantheon, and architecture, that echoed those of the kingdoms in Yemen (*49*, *50*). This period was key for the development of terraced agriculture in the highlands (*51*, *52*), as well as the formalization of trade with the interior of the continent, and with Arabia and northeast Africa, via the ports of the Red Sea (*53*).

Mobility as a result of colonization, conquest, and conflict is evidenced throughout this period, as is mobility for maritime and overland trade and the maintenance of social ties (Fig. 2D). The period saw many innovations, including the introduction of camels, horses, and other pack animals (*54*), and the development of chariots and sailing craft. During this period, mobility and what motivated it changed considerably with polities increasingly controlling territory, resources, and people. The importance of climate and environment, of resource availability, and of subsistence strategies decreased in a very short time as a result of shifting human-environment and social relations.

### Human movement in Inner Eurasia

**Fig. 3.**
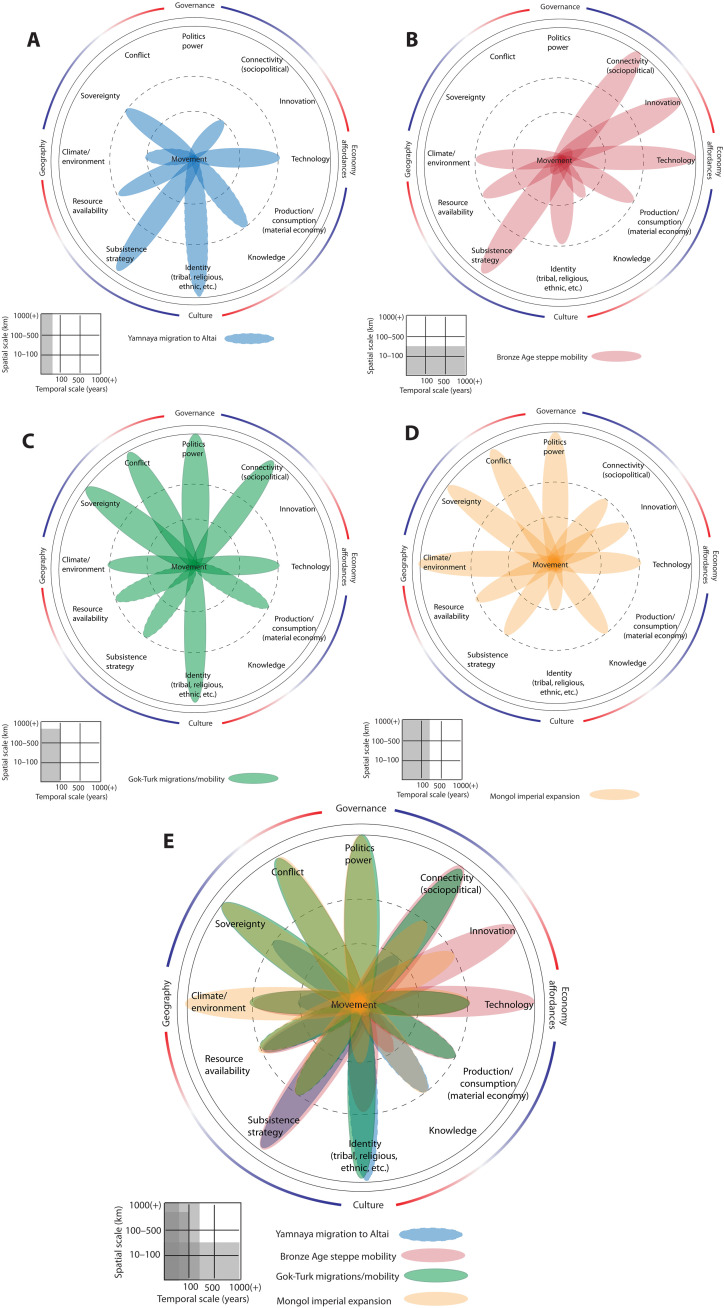
Dahliagram analyses of human movement in Inner Eurasia. (**A**) Yamnaya long-distance migration [~5000 to 4900 before present (B.P.)], (**B**) middle Bronze Age steppe herders (~4200 to 3200 B.P.), (**C**) early Turkic expansion (6th to 7th centuries CE), (**D**) Mongolian expansion (~13th to 14th centuries CE), and (**E**) multiperiod composite dahliagram for Inner Eurasia.

Population movement has played a formative role in shaping societies of the Inner Eurasian steppes from prehistory to the present (*55*, *56*). Whether one considers long-distance migratory episodes, historical invasions, plague-related population displacements, or durable ecological patterns of seasonal mobility, archaeology and history together document the paramount role of human movement on the ideological, economic, and political geography of the Eurasian steppe. Four key phases of human movement in Inner Eurasia include the early and middle Bronze Ages, the Turkic era, and the Mongol era (Fig. 3).

Human movement in the context of the Eurasian steppe has always been respondent to both immediate and long-term human-environment interactions (*57*, *58*), operating at various geographic scales. The seasonal mobility patterns of most Eurasian pastoralist communities, for example, are heavily influenced by the availability of pasture, water, and other climatically responsive environmental resources, used to support their agro-pastoralist economies (*59*). However, the two prehistoric cases considered here—an early Bronze Age long-distance migratory event and pastoralist mobility patterns of the middle Bronze Age—differ in temporal longevity, in spatial extent, and in the combination of push/pull factors underpinning them.

The ancient genomic record of Eurasia documents a close ancestral tie between west Eurasian “Yamnaya” populations and Altaic “Afanasievo” communities separated by thousands of kilometers, suggesting a rapid, long distance eastward migration by ancient herders at the start of the 5th millennium B.P. (*60*–*62*). Subtle differences in the archaeological remains between regional communities (among the Yamnaya) and the migrants to the Altai suggests complex intracultural social dynamics that may have prompted periodic out-migration among select Yamnaya groups, both westward and eastward (*63*). Whether impelled by social or ideological drivers, Yamnaya migration was partly facilitated by new technologies such as bullock carts and novel innovations such as horse riding and dairying (*64*, *65*). There is little corresponding zooarchaeological evidence for domesticated horse remains in Altaic Afanasievo settlements, perhaps indicating that horse riding was not overly influential in long-distance migration at this time, despite its potential (*65*, *66*). Regardless, we must assume that a considerable degree of transregional knowledge and expertise was needed to navigate the vast steppe environment, whether moving by foot, by cart, or by horse. Paleoclimatic evidence for environmental amelioration suggests slightly improved pasture quality at this time in prehistory, but early Bronze Age herders had long established a resilient adaptive strategy to drought and other climatic stressors (*67*). Therefore, when we consider the wider array of factors in comparative perspective, the cumulative picture suggests that the Yamnaya migration to the Altai was likely prompted by conditions related to intergroup social dynamics, and predicated upon aspirations of tribal or political sovereignty among communities within the wider Yamnaya social arena, rather than being an adaptive response to changing environmental conditions or sparked by new technology per se (Fig. 3A).

Roughly 1000 years later, regionally specialized mobility patterns among middle Bronze Age pastoralists reflect a significantly more localized geographic scale and character (Fig. 3B). Eurasian pastoralists of the 4th millennium B.P. had engineered ecologically well-adapted, multispecies (agro-)pastoralist subsistence strategies (*68*) and honed their mobility patterns to exploit both steppe and mountain ecologies (*69*, *70*). This era was also heavily influenced by the growth of large-scale trade networks (*71*–*73*), which was not a predominant stimulus for the earlier Yamnaya migration. Widespread growth and transfer of technological innovation such as tin-bronze metallurgy, horse riding, and grain farming each played a heightened role on mobility as they drew communities into regional arenas of trade and exchange, and facilitated forays into novel environments (*74*, *75*). Although paleoclimatic archives illustrate a broad climatic trend toward cooler, dryer conditions, there is little to suggest an abrupt or large-scale environmental impulse that would have forced more extensive mobility after 4000 B.P. (*76*). Rather, human movement across (and within) Eurasia in the fourth millennium B.P. appears to be influenced most heavily by novel technologies and network expansions for trade and interaction that were nascent in earlier millennia (*77*).

Historically documented Central Asian migrations have been discussed for centuries in relation to Huns, Avars, and other peoples that appeared suddenly on the borders of the Roman empire and later hegemonies of Europe (*78*). Imperial formations established by the 6th century CE in the eastern part of Eurasia reflect a clear configuration of the forces underlying population movement, as political and economic transformations of the Iron age evolved to shape diverse modalities of mobility up to the present (Fig. 3C). The end of the Eastern Türk empire and dispersal or relocation of Turkic peoples in eastern Inner Eurasia have been connected with volcanic events that further intensified the general cooling observed during the Late Antique Little Ice Age (*8*).

Environmental aspects and, in particular, the availability of abundant pasture were essential to the imperial movement of pastoral, horse-based people and their armies. Population scale and the force of imperial warfare in the Mongol period (Fig. 3D) placed increased stress on environmental resources, exacerbating the impact of regional climate change evident across the eastern and central Eurasian steppe zone (*79*, *80*). Yet, comparatively, mobility on the part of the Turks, Uyghurs, and Mongols was likewise influenced by nuanced differences in trade possibilities and commercial connectivity. The Uyghurs’ control of the silk trade (6th to 8th century CE), for example, likely had greater relevance to their mobility, whereas conflict can be considered as more influential in the Mongol imperial movements. The dissolution of the Uyghur empire, which also led to the relocation of nomads from Mongolia to northern China and to the Tarim Basin, in today’s Xinjiang, may also have been due to long-term climatic variation in combination with political transformation (*81*).

For Turko-Mongol polities, centralized political organization and social structures, including kinship, ethnic, and class stratification, drove their imposition of sovereignty over conquered lands. Their mobile lifestyle and associated technology (such as tents mounted on carts or collapsible tents carried on pack animals) also facilitated mobility. Continuous conflicts and factional struggles developing within the Türk and Mongol empires contributed to movement, together with policies, in the Mongol empire, that aimed to remove and relocate entire population groups (*82*).

While the comparative spatial scale of population movement underlying Turkic and Mongol imperial expansion was restricted or enhanced by the availability of environmental resources (grasslands) in Eurasia, there were notable differences in terms of their previous knowledge and of the speed and temporal range of the movement. Türkic polities advanced throughout territories that were largely unknown to them; the long-term effects of their movements include a general expansion of Turkic languages across Central Asia and southwest Asia and into Anatolia. On the contrary, the Mongols likely had prior information about at least some lands and tended to incorporate within their rank’s large numbers of conquered peoples—including Türks (broadly defined). Demographic patterns based on genetic analysis seem to indicate a higher-than-normal reproduction rate of Mongols across Eurasia (*83*), which, however, did not result in large-scale linguistic changes but rather fostered cultural assimilation. Climatic elements such as droughts or cooler periods have also been identified as possible push factors (*84*).

While timescales, territorial extension, and historical relevance of the two movements are different, the expansions of both Türk and Mongol empires have left indelible traces on the Inner Eurasian linguistic, cultural, political, and genetic map. Their forms of movement cannot be simply associated with climatic factors but need to be considered in relation to the more general forms of mobility underlying nomadic ecology and their dynamic political-economic systems. The respective dahliagrams for each era expose the nuanced balance of factors that need to be considered at any given time in light of their specific archaeological, paleoenvironmental, and historical circumstances.

### Human movement across the North Atlantic

Human movement across the North Atlantic has been an ongoing process for more than 1000 years. As early as the late 10th century CE, Vikings expanded across the North Atlantic and even became the first Europeans to reach (present day) Newfoundland and establish a small and short-lived settlement (L’Anse aux Meadows, 1021 CE) (*85*).The Vikings reached Newfoundland from Greenland, settling there in 985 BCE through an expedition from Iceland. Estimates suggest that the number of settlers ranged between 500 and 1500, split between “Eastern” and “Western” settlements. Their population apparently grew rapidly and peaked around the middle of the 12th century CE, with up to 3000 people in both settlements combined (*86*, *87*).

Viking expansions were generally favored by conditions of the Medieval Climate Anomaly, which created significantly warmer summers around the year 1000 (*88*). Moreover, the fate of the Greenland Norse has also been linked with the cooling that followed later, in a transition to the Little Ice Age (LIA). A first phase of cooler temperatures set in after 1250. From 1400 CE, the cooling became more marked and sustained. The western settlement was depopulated between 1350 and 1400, and the eastern settlement was abandoned around 1450 CE. In the 1990s, synchronicity with climate change combined with archaeological evidence established a narrative, according to which the “collapse” of the Greenland Norse was a case of ecological overshoot and maladaptation (*89*, *90*). Supposedly, they had clung on to field farming, to increasing the size of their herds, and to a meat-based diet for too long and, thus, missed the opportunity to successfully adapt to changing conditions.

However, this narrative has not withstood the test of time. Analyses of carbon isotopes in human bone remains have shown that the proportion of marine proteins increased steadily between the 11th and 15th centuries CE. The Greenland Norse had increasingly covered their needs from marine hunting and also adapted their agrarian strategies (*91*, *92*). In sum, the Greenland Norse did not fail to adapt to the challenges posed by the transition from a warmer to a cooler climate. If at all, they failed in spite of their adaptative resiliency.

**Fig. 4.**
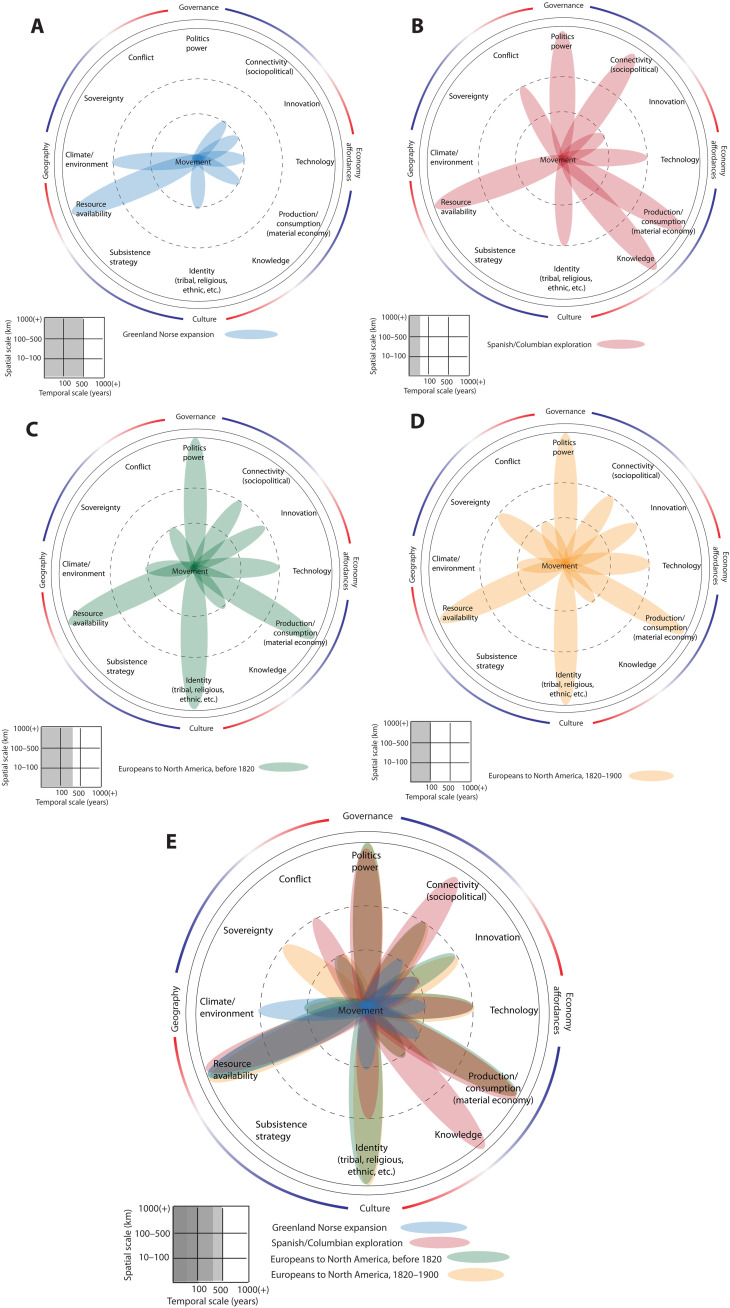
Dahliagram analyses of human movement in the North Atlantic region. (**A**) Greenland Norse expansion (~1000 to 1400 CE); (**B**) Spanish/Columbian exploration (late 15th to 16th century CE); Pre-1820 European Migration (17th to early 19th centuries CE); (**D**) post-1800 European migration (19th century CE); and (**E**) multiperiod composite dahliagram for the North Atlantic.

Even more important than such reinterpretations is the recent discovery that the economic motivation for expansion to Greenland came from Viking involvement in the European ivory trade (Fig. 4A) (*93*). Their walrus hunting grounds shifted further north from Icelandic waters and as far east as the coast of Newfoundland. Thus, settling in precarious places, they had aims quite different from pushing agriculture to the limits. They simply followed a lucrative prey and, in their efforts, they were temporarily favored by relatively warm conditions. However, the European ivory trade phased out. Elephant tusks gradually flooded the market and put the prices for ivory from North Atlantic walrus hunting under severe pressure. It is now widely believed that the Greenland Norse did not await death in their settlements but simply abandoned them and returned to Iceland for more productive economic endeavors.

After 1492, entirely new routes of mass migration emerged in the Atlantic world (*94*). The accidental “discovery” by Christopher Columbus of land masses hitherto unknown to Europeans fueled desires among rulers, explorers, and conquerors to extract gold, silver, and other natural resources (Fig. 4B). Mining and, soon afterward, cash crops created a demand for labor among Spanish and Portuguese imperial forces initially met by enslaving indigenous peoples. When natives died in great numbers from diseases and violence, deportation of human labor from Africa sparked one of the most pronounced eras of forced human movement in human history (*95*, *96*).

First, the Spanish and Portuguese empires and then the French and British empires in the Americas were erected on the economic foundations of plantations and a demand by markets in Europe and Asia for their products (*97*). All highly profitable cash crops—tobacco, indigo, sugar cane, etc.—required tropical or subtropical conditions and could not be grown in Europe. Hence, climatic zones and plant geography were inseparably intertwined with economies in colonial America (*98*–*100*). This plantation economy drove transatlantic movements of both African slaves and Europeans, the latter crossing the Atlantic as indentured servants. Nevertheless, most of the European emigrants made free choices about their movements, while this was not the case for enslaved Africans. For this reason, the African Slave trade must be modeled as a separate case and demands a more nuanced consideration than space here allows. The dahliagrams presented here for the North Atlantic therefore focus on emigration from Europe to the Americas, in the 15th and 19th centuries, respectively.

Estimates for European transatlantic migration after 1492 help identify two distinct periods (Fig. 4, C and D): Before century 1820, numbers were low, while they went up rapidly afterward. Only in the latter period did Europeans outnumber forced migrants from Africa. Over the long term, the availability of arable land and labor remained important pull factors, while population growth in Europe became a dominant push factor during the 19th-century industrial transition, when in some parts of Europe, such as the German territories (before 1871), industrial labor struggled to keep up with demand (*101*). Chain migration (connectivity) was a common phenomenon already in the 18th century. People regarded themselves as members of a group—a nation, a religious community, or family, etc.—both as emigrants and immigrants. Religious and other forms of identity became key motives for leaving one place and for choosing another. The industrial transition saw major technological innovations (e.g., railway and steam boats), which made long-distance migration generally more affordable and, thus, contributed to the rise in numbers.

Only few authors have considered the role of the LIA in North Atlantic migration and assessed that it “had a major impact” due to cases of harvest failure (*102*, *103*). However, the influence of LIA fluctuations on agrarian production remains controversial among economic historians (*104*, *105*). Attributing meteorological events to the LIA is just one of the difficulties (*106*). On the other hand, evidence for climatic anomalies causing crises in agrarian production is overwhelming. Events such as the “Great Frost” in 1740 affected large parts of Europe and can be linked with emigration in some cases, e.g., from Ireland (*107*). In the 19th century (Fig. 4D), Irish emigration to the United States peaked after the Great Famine of 1846–1849. Although a potato blight was its proximate cause, neither the famine nor emigration can be reduced to environmental factors. The Irish and Germans were the two largest groups of immigrants to the United States after 1820, followed by the British. German immigration peaked in 1816–1817, 1846–1857, 1864–1873, and 1880–1893 (*108*). Some of these peaks [1816–1817 and 1846; (*109*)] coincided with bad weather, harvest failures, and high prices for cereal crops. Others were dominated by political motives (after the failed Revolution of 1848). While agrarian crises did form a recurring pattern in large parts of Europe over the entire period of transatlantic migration until 1900, their overall contribution can hardly be assessed quantitatively. Parts of France, the Dutch Republic, Switzerland, or the Austrian Empire were more or less equally affected by climatic anomalies and extremes, often at the same time, and yet, they did not produce the same peaks in the amounts of people that departed for the New World. The importance of climatic and/ or (other) environmental circumstances varied in time and place, and they were always intertwined with political, social, and economic factors.

## DISCUSSION

**Fig. 5.**
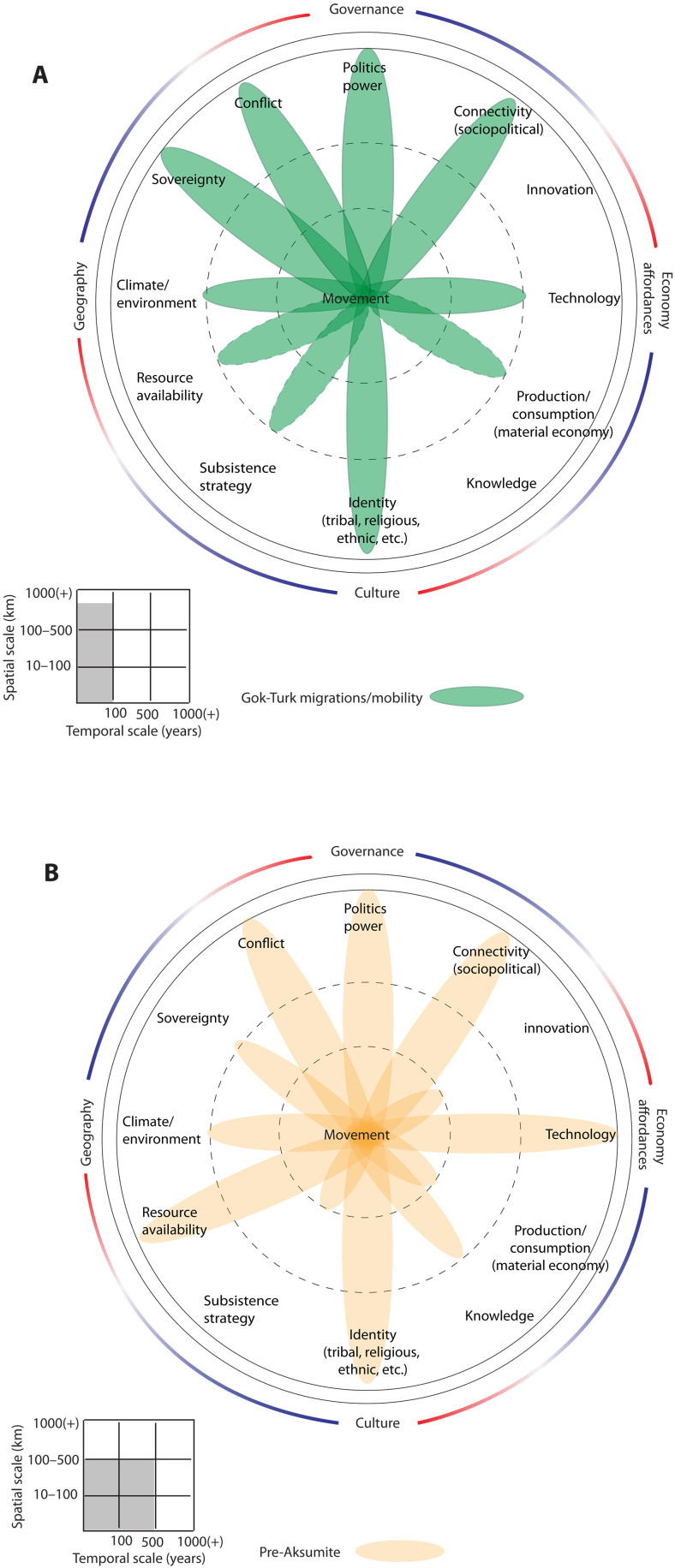
Comparative dahliagrams of emerging empires show similar driving factors for human movement. (**A**) Early Turkic expansion (6th to 7th centuries CE). (**B**) Pre-Aksumite period [~2800 to 2050 before present (B.P.)].

Given what is known about the diverse factors that shape human behaviors such as population movement (*21*), the dahliagram facilitates multidisciplinary researchers to correlate their knowledge and data in a paradigmatic fashion while remaining agnostic to incongruent units of measure, data scale, resolution, and source. The tool remains pliable and adaptable to the specific needs of a given research design. So too, it exposes domains of knowledge that are underdocumented in paleoclimatology, archaeology, or history or which simply have not been considered directly as factors in shaping behaviors like population movement. Hence, it requires the user to think beyond simply causality and consider the intersections of social, economic, political, and environmental conditions for each case. The resulting visualizations further serve to expose previously unexpected parallels among different regional cases that might otherwise never be viewed side by side and therefore open the possibility of multiregional comparative analysis. Through our exercise of modeling mobility in both the early Turkic era of Eurasia and the pre-Aksumite period of eastern Africa, for example, the resulting dahliagrams chart highly comparable driving factors, with only minor shifts in the magnitude of impact across a few domains (Fig. 5).

Although these cases are separated historically by hundreds of years and situated thousands of kilometers apart, population movement within these emergent empires appears to have been rooted in similar forces of political and social identity, as well as ambitious interests to acquire regional resources and stimulate trade and connectivity. Environmental factors were an omnipresent concern but appear to be outweighed by factors such as conflict and sovereignty. The historical implications of mobility within these formative empires in their respective era and region are unique, but only when visualized in the dahliagram do we see the shared correlations across an array of factors that may produce fruitful onward investigation into their behavioral similarities at the human-environmental nexus.

At first glance, any given dahliagram may seem subjective and idiosyncratic, built for a particular case through interdisciplinary consensus among a given team of specialists. However, we consider the dahliagram as a necessary rubric to establish common understandings of disparate analytical approaches and forms of evidential support. The efficacy of the dahliagram lies first and foremost in its capacity to spark dialogue and debate, because different research partnerships might generate alternative renderings of available data. Accordingly, we do not propose that any particular dahliagram illustrated above must provide the final analytical word in each case. Our goal, rather, is to demonstrate the efficacy of the dahliagram to translate disciplinary topics as well as empirical and qualitative data sources into a single visual illustration or historical time series that can be shared among researchers from different specializations and quickly communicated, understood, and incrementally modified.

The main stimulus to create such a tool came from our own experience with interdisciplinary collaborations as climate scientists, archaeologists, and historians. The technical complexities pertaining to each of these disciplines and deep epistemic cleavages between them, make approaches to the past fraught with uncertainties. The dahliagram visualization thus emerges as an interdisciplinary response to ongoing challenges of socio-environmental studies, allowing nuanced connections to be drawn between historical phases of climatic stress and the assumed rise and fall of human settlements, empires, and social cohesion (*110*).

Within the discipline of climate science similar qualitative visualizations have been produced for the express purpose of providing an immediate and synthetic representation of how the planet is being affected by anthropogenic climate change and environmental degradation. One tool, referred to as “planetary boundaries,” is used to translate diverse quantifications of atmospheric change, soil and water chemistry, and attendant environmental transformations in relation to sustainability “boundaries.” Planetary boundaries has been widely adopted as an effective way to communicate the risks and impact of human and natural forcing on the planet’s ecosystemic health and sustainability (*24*, *111*–*113*). The power of such tools lies in their capacity to promote scientific consensus around acceptable human influence on the environment and long-term climate dynamics.

Given its ease of use, we hope that the dahliagram will be adopted to stimulate further explorations of complex human behaviors by leveraging multidisciplinary team building and consensus, especially concerning studies of the past. As qualitatively and quantitatively higher-resolution data emerge, we envision the dahliagram evolving into a multidimensional tool whose applications could range from the design of a multidisciplinary research project to presentation of the results of an interdisciplinary study or conclusions of historical causality. The ultimate aim of much of contemporary research into human behavioral dynamics is to integrate diverse knowledge to advance our understanding of the interplay among social and environmental factors while not sacrificing data resolution or the critical lens of disciplinary experts. The dahliagram drives research toward an explicit consideration of concomitant factors and inspires us to think critically beyond pre- and misconceptions concerning causality in both historical and contemporary contexts.
